# *N*-Demethylsinomenine, an active metabolite of sinomenine, attenuates chronic neuropathic and inflammatory pain in mice

**DOI:** 10.1038/s41598-021-88521-z

**Published:** 2021-04-29

**Authors:** Zhiyong Zhou, Nanqing Qiu, Yuntao Ou, Qianqian Wei, Wenting Tang, Mingcong Zheng, Yaluan Xing, Jie-Jia Li, Yong Ling, Junxu Li, Qing Zhu

**Affiliations:** School of Pharmacy, Nantong University, 19 Qixiu Road, Nantong, 226001 Jiangsu Province China

**Keywords:** Drug discovery, Neuroscience

## Abstract

Chronic pain is a significant public health problem that afflicts nearly 30% of the global population, but current pharmacotherapies are insufficient. Previous report indicated that *N*-demethylsinomenine, an active metabolite of sinomenine, is efficacious against postoperative pain. The present study investigated whether *N*-demethylsinomenine is effective for chronic painful conditions or whether repeated treatment alters its effect. Both chronic constriction injury (CCI) surgery and complete Freund’s adjuvant (CFA) intraplantar injection induced significant and reliable mechanical allodynia at least for 7 days. Acute treatment with *N*-demethylsinomenine (10–40 mg/kg, i.p.) dose-dependently attenuated the mechanical allodynia both in CCI-induced neuropathic pain and CFA-induced inflammatory pain in mice. The potency of *N*-demethylsinomenine for reducing CFA-induced mechanical allodynia was slightly higher than sinomenine. During the period of repeated treatment, *N*-demethylsinomenine maintained its anti-allodynic effect against both neuropathic and inflammatory pain without producing carry-over effect. Pretreatment with bicuculline, a selective γ-aminobutyric acid type A (GABA_A_) receptor antagonist, almost completely blocked the anti-allodynia of *N*-demethylsinomenine (40 mg/kg) both in CCI and CFA-treated mice. Our findings indicated that *N*-demethylsinomenine exhibits GABA_A_ receptor-mediated anti-allodynic effects in mouse models of neuropathic and inflammatory pain, suggesting it may be a useful novel pharmacotherapy for the control of chronic pain.

## Introduction

Chronic pain is a pervasive public health problem and is estimated to affect up to 30% of the adults globally, which exerts a significant toll on society^1^. Analgesic drugs play a central role in the management of chronic pain. However, current analgesics widely used in clinical treatment are often limited by incomplete efficacy and side-effects^2^. For example, NSAIDs have limited effectiveness for inflammatory pain and are often ineffective for neuropathic pain. Although opioids are the most effective analgesics for acute pain, their use is limited by the high abuse liability, analgesic tolerance, and toxic effects in elevated doses. In addition, the use of other analgesics including antidepressants and anticonvulsants can be limited by unwanted effects such as sedation, dizziness, and memory deficits. In clinical practice, less than a third of patients with chronic pain have reported at least moderate pain relief and most patients continue to live with some level of pain irrespective of the treatment^3^. As such, there is a high need for developing new and more effective drugs for the treatment of chronic pain.

Sinomenine, the main bioactive ingredient isolated from traditional Chinese herb *Sinomenium acutum,* has been used in the treatment of rheumatoid arthritis clinically and it seems to be more efficacious and safer than NSAIDs^4^. Recently, sinomenine has been reported to have a wide spectrum analgesic effect in rodent models of nociceptive, inflammatory and neuropathic pain^5^. Subsequently, more evidence suggests that sinomenine is an effective analgesic agent for chronic pain including neuropathic and inflammatory pain, and several mechanisms mediating sinomenine-induced antinociception have been proposed^6^. For example, sinomenine is found to produce analgesic effect via a central mechanism in the anterior cingulate cortex by regulating the GluN2B NMDA receptors and mTOR signals in a mouse model of chronic inflammatory pain^7^; sinomenine may function as an inhibitor of P2X_3_ receptor in dorsal root ganglia to relieve the hyperalgesia in a mouse model of diabetic neuropathic pain^8^. Furthermore, our previous studies demonstrated that sinomenine exerts antinociceptive effects in rat models of neuropathic pain and postoperative pain via GABA_A_-mediated mechanism^9,10^.

*N*-Demethylsinomenine[(+)-4-Hydroxy-3,7-dimethoxy-7,8-didehydromorphinan-6-one], an *N*-demethylated product of sinomenine, has been identified as a major metabolite of sinomenine in pharmacokinetic studies^11,12^. In addition, *N*-demethylsinomenine is also a natural product isolated from the stems of *Sinomenium acutum* and displays protective effects against hydrogen peroxide-induced cell injury^13^. Our previous study demonstrated that *N*-demethylsinomenine has anti-allodynic effect in a mouse model of postoperative pain^14^. Here, we hypothesized that *N*-demethylsinomenine may also exert antinociceptive effects for chronic pain and have potential to be developed as a new analgesic agent for chronic pain.

In the present study, we investigated the antinociceptive effects of *N*-demethylsinomenine on two commonly-used mouse models of chronic pain, chronic constriction injury (CCI) induced neuropathic pain and complete Freund's adjuvant (CFA) induced inflammatory pain, and compared these effects with those of sinomenine. Because in chronic pain conditions pharmacological treatment requires repeated dosing which may produce antinociceptive tolerance, we also examined the antinociceptive effects during repeated treatment.

## Results

### Effect of acute treatment with *N*-demethylsinomenine on mechanical allodynia in mice induced by CCI surgery or CFA injection

Prior to the CCI or CFA procedure, the baseline PWT in mice in response to von Frey filament is nearly 1.4 g, which has no difference between the control group (received sham operation or saline plantar injection) and the model group (*P* > 0.05) and remained stable during a period of 7 days of repeated measures in control group. However, one day after CCI surgery or CFA injection, the PWT of the right hind paw markedly and significantly decreased to nearly 0.3 g and remained stable with repeated measures throughout the 7 days of tests, indicating significant and reliable mechanical allodynia (see supplementary information, Fig. S1). In contrast, the PWT of the left hind paw remained unchanged at the baseline level during repeated measurement (data not shown).

**Figure 1:**
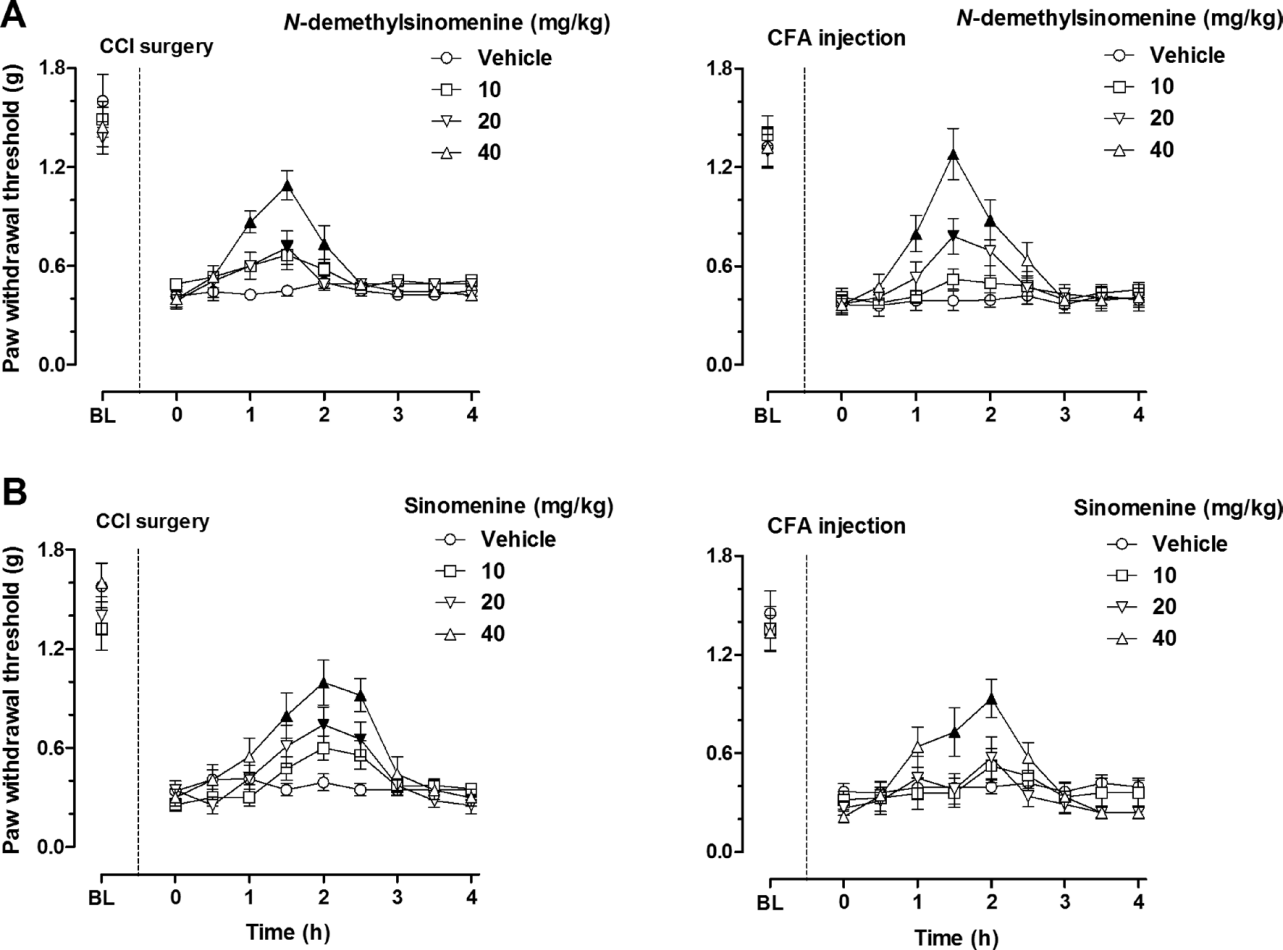
Effects of acute *N*-demethylsinomenine (**A**) and sinomenine (**B**) treatment in CCI-operated (left) and CFA-treated (right) mice. BL: baseline value prior to CCI surgery or CFA injection; Filled black symbols indicated significant difference in *N*-demethylsinomenine or sinomenine treatment group as compared to the vehicle-treated group. (n = 8–10 per group).

Acute treatment of *N*-demethylsinomenine dose-dependently increased the PWT of the right hind paw in the mice receiving CCI surgery or CFA injection (Fig. 1A). Vehicle treatment did not significantly change the PWT in mice during the 4-h testing period. *N*-demethylsinomenine treatment gradually increased the PWT and this effect reached the maximum at 1.5 h and then subsequently faded away at 2.5 h after drug administration of different doses (10–40 mg/kg) both in CCI- and CFA- treated mice. Two-way ANOVA and Post hoc analyses indicated similar results both in CCI and CFA treated mice that 20 mg/kg *N*-demethylsinomenine significantly increased the PWT at 1.5 h after treatment (*P* < 0.05) and 40 mg/kg *N*-demethylsinomenine significantly increased the PWT between 1 and 2 h after treatment (*P* < 0.001 or *P* < 0.05); however, treatment with 10 mg/kg *N*-demethylsinomenine did not markedly alter the PWT during the 4-h period of repeated measurement (*P* > 0.05). In comparison, we found that sinomenine treatment dose-dependently attenuated mechanical allodynia both in the CCI mice and the CFA mice and produced maximal effect at 2 h after drug administration (Fig. 1B). Two-way ANOVA and Post hoc analyses indicated that 20 mg/kg sinomenine significantly increased the PWT between 2 and 2.5 h after treatment as well as 40 mg/kg sinomenine significantly increased the PWT between 1.5 and 2.5 h after treatment in the CCI mice (*P* < 0.05 or *P* < 0.01). In the CFA model, only 40 mg/kg sinomenine significantly increased the PWT between 1.5 and 2 h after treatment (*P* < 0.05 or *P* < 0.01). These results indicate that these two drugs displayed similar duration of action (1 h) at the same dose level (40 mg/kg), although the onset of action for *N*-demethylsinomenine (1 h) seemed to be slightly faster than that of sinomenine (1.5 h), which may not always attain statistical significance. In addition, both drugs did not significantly alter the PWT of the left paw (non-injured paw) in mice during the testing period (data not shown). Importantly, selected doses (10–40 mg/kg) of *N*-demethylsinomenine or sinomenine did not alter the spontaneous locomotor activity in healthy mice, showing no sedation or motor impairment (see supplementary information, Fig. S2).

**Figure 2:**
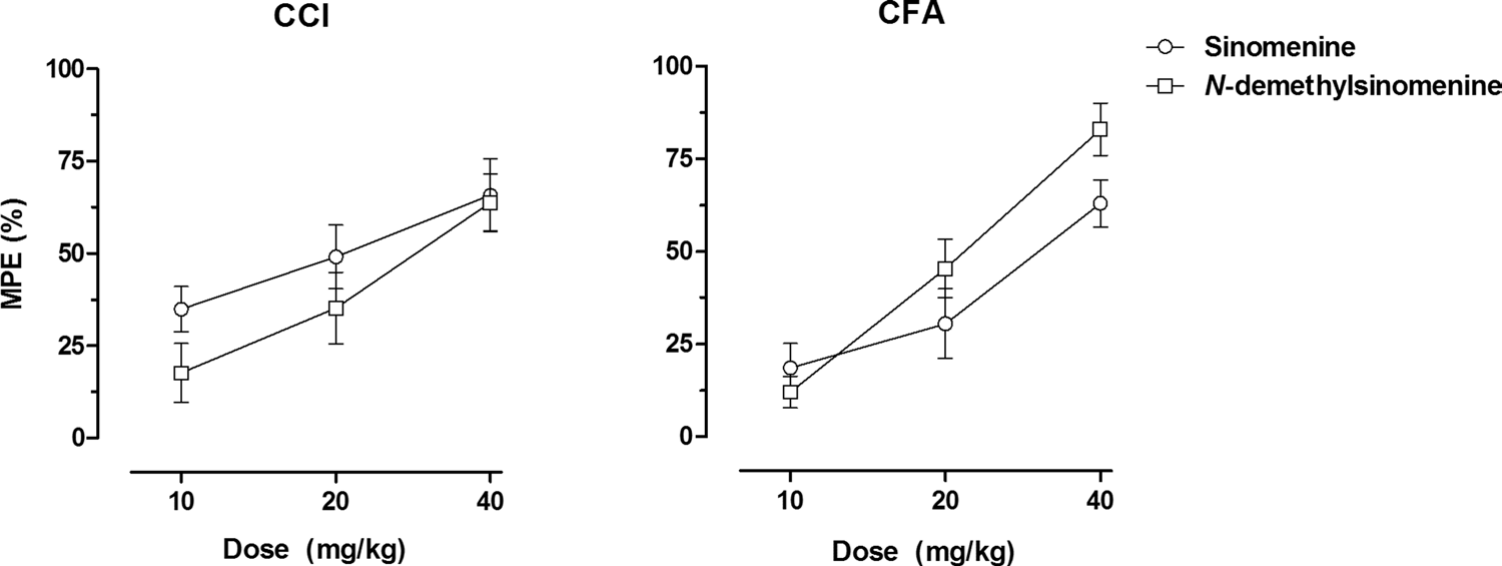
Dose–effect curves of sinomenine and *N*-demethylsinomenine in CCI-operated (left) and CFA-treated (right) mice. Data were expressed as percentage of maximal possible effect (% MPE) (mean ± S.E.M., n = 9–10 per group) and plotted as a function of drug dose; 100% MPE represented data from the normal baseline mechanical PWT before CCI surgery of CFA injection.

The anti-allodynic effectiveness of *N*-demethylsinomenine and sinomenine was compared by converting the data into the maximal possible effect (% MPE) and the dose–effect curves for these two drugs from the CCI and CFA models were shown in Fig. 2. In CCI model, *N*-demethylsinomenine and sinomenine produced a maximum of 63.7% MPE and 65.7% MPE, respectively, showing similar effectiveness. In contrast, the maximal effect produced by 40 mg/kg *N*-demethylsinomenine (83.0% MPE) was higher than that produced by 40 mg/kg sinomenine (63.0% MPE) in CFA model mice. Nonlinear regression analyses revealed that the dose–effect curves of *N*-demethylsinomenine and sinomenine for CCI model were not significantly different from each other and can be fitted with one curve (F[2, 50] = 1.82, *P* > 0.05). Meanwhile, the ED_50_ values (95% confidence limits, CL) of *N*-demethylsinomenine and sinomenine were 28.4 (20.5, 39.4) mg/kg and 20.1 (12.9, 31.5) mg/kg for CCI model, respectively, with no significant difference between each other. However, for CFA model, the dose–effect curve of *N*-demethylsinomenine was different from that of sinomenine, and the ED_50_ values (95% CL) of *N*-demethylsinomenine [21.5 (18.2, 25.5) mg/kg] was lower than that of sinomenine [30.1 (22.3, 40.5) mg/kg] (F[2, 50] = 3.26, *P* = 0.046). Thus, *N*-demethylsinomenine seems to be more potent than sinomenine in reducing CFA- but not CCI-induced mechanical allodynia.

### Daily treatment with *N*-demethylsinomenine maintained its anti-allodynic effect in mice

**Figure 3:**
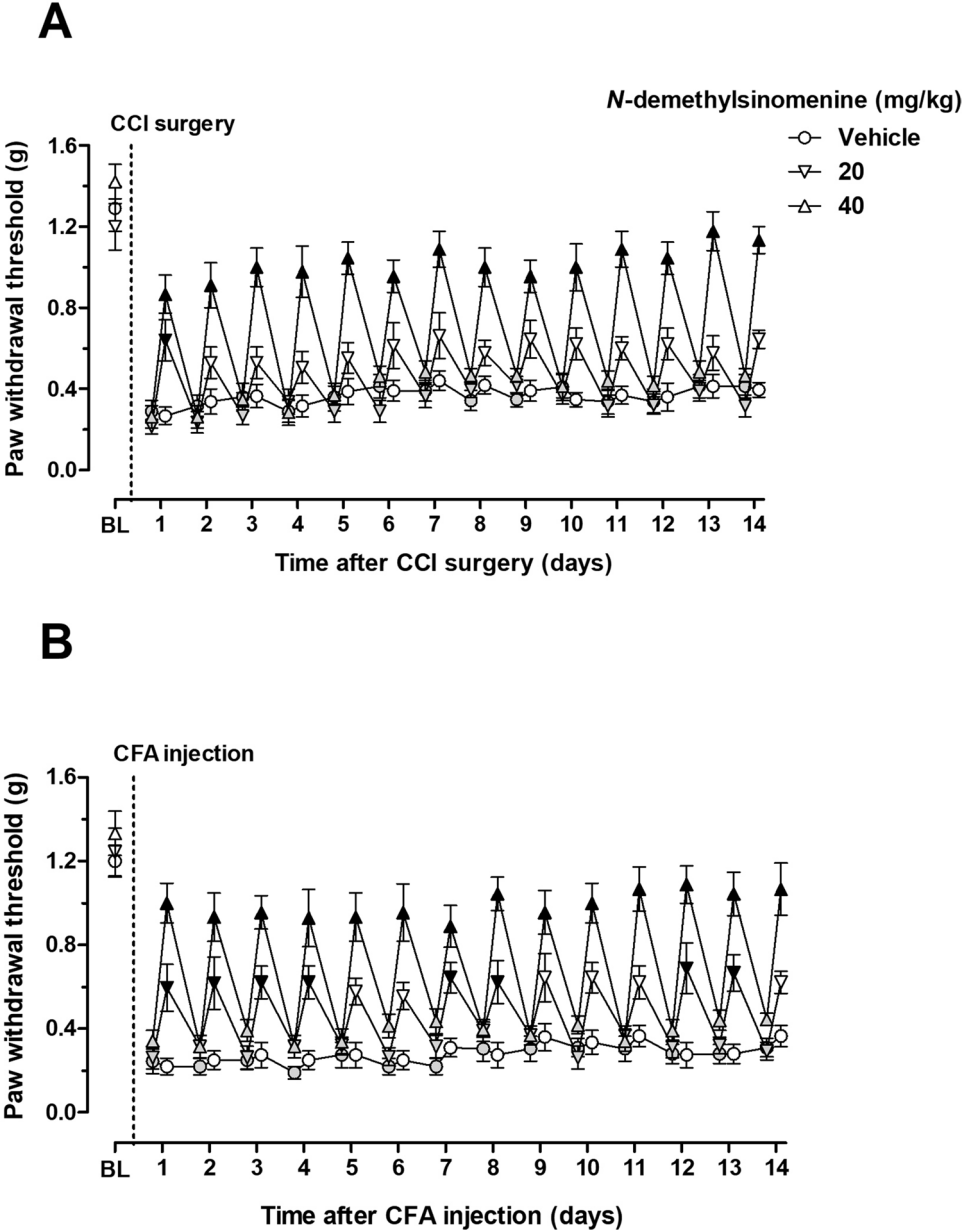
Effects of repeated *N*-demethylsinomenine treatment in CCI-operated (**A**) or CFA-treated (**B**) mice. Data were expressed as mean ± SEM (n = 8–9 per group), assessed by two-way ANOVA with repeated measures followed by Bonferroni post hoc analysis. BL: baseline value prior to CCI surgery or CFA injection; Filled grey symbols indicated the data prior to administration of *N*-demethylsinomenine each day during the period that mice received daily treatment. Filled black symbols indicated data significantly different from the corresponding vehicle group (*P* < 0.05).

Daily treatment with *N*-demethylsinomenine dose-dependently attenuated mechanical allodynia both in CCI-operated and CFA-treated mice and maintained this anti-allodynic effect throughout the period of 14 days of daily treatment (Fig. 3). Two way ANOVA and Post hoc analyses revealed that daily treatment with 20 mg/kg *N*-demethylsinomenine improved the mechanical allodynia at 1 day after CCI surgery (*P* < 0.01) and daily treatment with 40 mg/kg *N*-demethylsinomenine attenuated the mechanical allodynia over a 14-day period of daily treatment after CCI surgery (*P* < 0.001). Daily 20 mg/kg *N*-demethylsinomenine administration increased the PWT between 1 and 4 days and at 7, 8, 12, 13th day after CFA injection (*P* < 0.05 or *P* < 0.01) and daily 40 mg/kg *N*-demethylsinomenine treatment increased the PWT throughout the period of 14 days after CFA injection (*P* < 0.001). In order to examine if *N*-demethylsinomenine had carry-over effect, the PWT was also measured prior to drug administration each day during the period that mice received daily treatment (filled grey symbols). In this case, the PWT prior to daily *N*-demethylsinomenine treatment (20 and 40 mg/kg) in each day was not significantly different from vehicle (saline) treatment both in the CCI and the CFA mice. Daily *N*-demethylsinomenine treatment did not carry over 24 h later and also had no *bona fide* curative effect on CCI-induced neuropathic pain and CFA-induced inflammatory pain.

### The effects of different receptor antagonists on the anti-allodynic effect of *N*-demethylsinomenine

**Figure 4:**
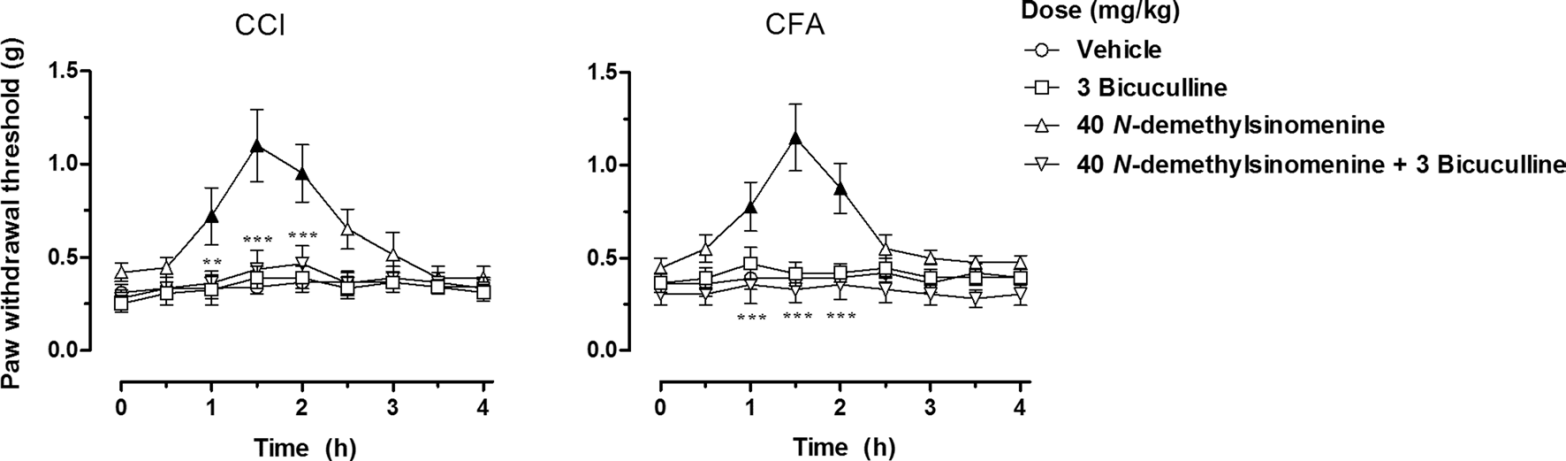
Pretreatment with GABA_A_ receptor antagonists bicuculline blocked the anti-allodynic effects of *N*-demethylsinomenine (40 mg/kg) in CCI-operated (left) and CFA-treated (right) mice. Filled black symbols indicated data significantly different from the vehicle-treated group (*P* < 0.05). **P < 0.01, ***P < 0.001 indicated significant differences between *N*-demethylsinomenine (40 mg/kg) alone and in combination with bicuculline group (n = 8 per group).

As shown in Fig. 4, pretreatment with 3 mg/kg bicuculline completely blocked the anti-allodynic effects of 40 mg/kg *N*-demethylsinomenine both in CCI-operated and CFA-treated mice, although it alone did not change the mechanical hypersensitivity. Two-way ANOVA and Post hoc analyses showed similar results both in the CCI and the CFA models that pretreatment with 3 mg/kg bicuculline significantly decreased the elevated PWT between 1 and 2 h after the administration of 40 mg/kg *N-*demethylsinomenine to pre-drug level, suggesting significant antagonism of *N-*demethylsinomenine-induced antinociception. We also examined if other receptors, such as opioid and 5-HT_1A_ receptor, mediated anti-allodynic effects induced by *N*-demethylsinomenine. However, we found that pretreatment with 1 mg/kg WAY100635 (serotonin 5-HT_1A_ receptor antagonist) or 2 mg/kg naltrexone (opioid receptor antagonist) did not significantly alter the anti-allodynia induced by an effective dose of *N*-demethylsinomenine (40 mg/kg) both in CCI and CFA model mice (see supplementary information, Fig. S3).

## Discussion

The primary findings of the present study were that *N-*demethylsinomenine, an active metabolite of sinomenine, attenuated the mechanical allodynia in mouse models of neuropathic and inflammatory pain. More importantly, these effects could be maintained throughout the period of repeated dosing. In addition, GABA_A_ receptors at least partially mediated *N*-demethylsinomenine-induced anti-allodynia under chronic pain conditions, which suggests that *N*-demethylsinomenine and its parent drug sinomenine may share similar pharmacological mechanisms^9^. Taken together, these data extended the previous finding that *N-*demethylsinomenine is effective against postoperative pain^14^ to showing that it is an efficacious analgesic for the control of chronic pain such as neuropathic and inflammatory pain.

Management of chronic pain is a tough task in clinical practice and current treatments such as NSAIDs and opioids are inadequate^15^. The need for developing effective and safe analgesics remains high. Sinomenine, the major bioactive ingredient of Chinese traditional herb medicine *Sinomenium acutum*, has been reported to have immunosuppressive and anti-inflammatory activities and is now available in clinical practice for the treatment of rheumatoid arthritis^4,16^. Recently, accumulating evidence indicate that sinomenine is an effective analgesic agent for the control of chronic pain^6^. *N-*demethylsinomenine, the major metabolite of sinomenine^12^, was reported to have anti-allodynic effect in a mouse model of postoperative pain by our previous study^14^. Although postoperative pain has a well-defined course and is different from chronic persistent pain conditions, the pathophysiological mechanisms mediating postoperative pain include both inflammatory and neurogenic components^17^. Thus, we hypothesized that *N-*demethylsinomenine could be effective for both neuropathic and inflammatory pain. CCI surgery has been widely used for developing a rodent model of nerve injury-induced peripheral neuropathic pain^18,19^. Intraplantar injection of CFA to unilateral hind paw has been proven to induce a typical rodent model of chronic inflammatory pain which develops long-lasting inflammation along with pain hypersensitivity^7,20^. In the present study, we applied the mouse models of CCI-induced neuropathic pain and CFA-induced inflammatory pain to investigate the antinociception of *N-*demethylsinomenine. Our data were consistent with the literature that one day after CCI surgery or CFA injection mice had developed significant mechanical allodynia and remained relatively stable throughout the experimental period of 7 days. According to these data, subsequent acute treatment studies were performed 24 h after CCI surgery or CFA injection. We found that acute *N-*demethylsinomenine treatment attenuated the mechanical allodynia both in these two chronic pain models with a dose- and time-dependent manner. Because the doses studied here (10–40 mg/kg) did not significantly reduce the spontaneous locomotor activity (Fig. 3), we considered that the improvement of pain hypersensitivity was not attributed to nonspecific behavioral suppression such as sedation or motor impairment. Meanwhile, sinomenine was used as a positive control and the comparison was made for the effects of *N-*demethylsinomenine and sinomenine. Consistent with previous reports^7,9^, we also found that sinomenine significantly ameliorated the mechanical allodynia in CCI- or CFA-induced pain models in mice, showing benefits for the control of chronic pain. When we compared the duration of actions between these two drugs, it seemed that the onset of action and the time to reach peak effect for *N-*demethylsinomenine are both slightly quicker than those for sinomenine, although these two drugs showed similar duration of action (Fig. 1), which is consistent with our previous report^14^. The comparison was also made for the dose–effect curve between these two drugs. We found that in the CFA model, the maximal effect (83.0% MPE) for *N-*demethylsinomenine was higher than that (63.0% MPE) for sinomenine at the same dose level (40 mg/kg), and the ED_50_ value (95% CL) of *N*-demethylsinomenine [21.5 (18.2, 25.5) mg/kg] is lower than that of sinomenine [30.1 (22.3, 40.5) mg/kg], which suggests that *N-*demethylsinomenine is more potent than sinomenine for attenuating CFA-induced mechanical allodynia. However, these two drugs showed similar potency for reducing CCI-induced mechanical allodynia as there is no statistical difference in the maximal effect and ED_50_ values for anti-allodynia between them in the CCI model. CFA-induced inflammatory pain is primarily driven by the production of inflammatory mediators in peripheral tissues and subsequently central sensitization^15^. We speculated that *N-*demethylsinomenine may have stronger anti-inflammatory activity than sinomenine, which may partially explain the phenomenon that *N-*demethylsinomenine is more effective than sinomenine in CFA-induced inflammatory pain in the current study. This speculation requires further studies to confirm.

Chronic pain is usually characterized as a persistent pain condition that lasts for more than 3 months. Management of chronic pain most likely needs repeated dosing, which may result in analgesic tolerance. For instance, documents reported that daily treatment with opioids such as tramadol and morphine at effective dose levels for 4 to 7 days is sufficient to produce significant antinociceptive tolerance^21,22^. Our previous report indicated that repeated *N-*demethylsinomenine treatment did not develop apparent drug tolerance in a mouse model of postoperative pain^14^. Therefore, we examined whether daily *N-*demethylsinomenine treatment could lead to the alteration of its anti-allodynic effects in chronic neuropathic and inflammatory pain. In the present study, daily treatment with *N*-demethylsinomenine dose-dependently attenuated mechanical allodynia and did not show a trend of anti-allodynic tolerance throughout the period of 14 days of daily treatment both in the CCI and the CFA models. Thus, it seems unlikely that the effect could change during prolonged *N*-demethylsinomenine use for the management of chronic pain. It should be noted the limitation in this study is that treatment with *N*-demethylsinomenine once daily leads to insufficient drug exposure every day due to its relatively short duration of action. Future study on the trend of tolerance development will apply the dosing strategies that mimic clinical practice more closely such as dosing to mice 2 to 3 times daily or using controlled or slow release formulations to achieve around-the-clock antinociception, which can better address the issue of antinociceptive tolerance^23^. It was also found that daily treatment with *N*-demethylsinomenine (20 and 40 mg/kg) had no carry-over effect from the previous treatment to the next, consistent with the finding that the effect of acute *N*-demethylsinomenine treatment almost completely dissipated 3 h after administration. These findings suggested that repeated *N*-demethylsinomenine treatment is a beneficial strategy for the control of chronic pain.

Spinal GABAergic neuron plays a pivotal role in modulating the process of pain signal^24^. The reduction of GABA_A_ receptor–mediated inhibition has long been considered as a critical player in spinal sensitization of painful behaviors^25,26^. Our previous report indicated that the anti-allodynic effect of *N*-demethylsinomenine against postoperative pain is at least partially mediated through GABA_A_ receptors^14^, which shows similar pharmacological mechanism to its parent drug sinomenine^9,10^. So, in the present study we investigated whether GABA_A_ receptor also mediates the anti-allodynic effect of *N*-demethylsinomenine on neuropathic pain and inflammatory pain via using a selective GABA_A_ receptor antagonist bicuculline. Here, we found that a large dose of 3 mg/kg bicuculline, which is sufficient to block the agonism of GABA_A_ receptor as reported previously^14,27,28^, completely reversed the anti-allodynic effect of *N-*demethylsinomenine against neuropathic and inflammatory pain, suggesting that GABA_A_ receptor plays a critical role in *N-*demethylsinomenine induced anti-allodynia. It is noted that whether *N-*demethylsinomenine produces antinociception by functioning as a direct GABA_A_ receptor agonist or indirectly enhancing GABAergic inhibition remains unclear. It requires further studies to confirm this notion.

Some reports showed that opioid receptor mediates the sinomenine-induced antinociception in mice^29,30^. Because *N*-demethylsinomenine is the active metabolite of sinomenine in vivo, the potential involvement of opioid receptor in *N*-demethylsinomenine induced anti-allodynia was examined via using a large dose of 2 mg/kg naltrexone (non-selective opioid receptor antagonist) that is enough to occupy the majority of opioid receptor as reported previously^31,32^. The descending monoamine pathway, especially the serotonin (5-hydroxytryptamine, 5-HT) system, plays an important role in endogenous pain modulation^33,34^. In addition, high-efficacy 5-HT_1A_ receptor agonists such as F13640 can produce intra- and postoperative analgesic effect^35^. Because sinomenine can attenuate CUMS-induced NE and 5-HT reduction in the mouse brain^36^. So, a dose of 1 mg/kg 5-HT_1A_ receptor antagonist WAY100635, which sufficiently blocks 5-HT_1A_ receptors^19,37^, was used to investigate whether 5-HT_1A_ receptor mediates *N*-demethylsinomenine-induced anti-allodynia. However, we found that pretreatment with WAY100635 or naltrexone dose not attenuate the anti-allodynic effect of *N*-demethylsinomenine, suggesting that both 5-HT_1A_ and opioid receptors are not involved in *N*-demethylsinomenine-induced anti-allodynia. These results are completely similar to the findings in our previous investigations on the pharmacological mechanisms of sinomenine^9^.

Previous reports showed that sinomenine has some adverse reactions, such as allergic reactions, gastrointestinal reactions, and circulatory systemic reactions, due to its effect on histamine release^38^. We also found that sinomenine at moderate to large doses can rapidly cause marked swelling of the paws in rats and gradually subsides. In addition, sinomenine at large dose (80 mg/kg) can induce sedative effect. In contrast, *N-*demethylsinomenine even at large dose of 80 mg/kg has no sedation and does not induce allergic reactions such as swelling (data not shown). In addition, *N*-demethylsinomenine is more potent than sinomenine for attenuating CFA-induced mechanical allodynia as shown in the present study. Together, it seems that *N*-demethylsinomenine may produce more powerful analgesic effect and less adverse reactions than sinomenine, suggesting the benefits of using *N*-demethylsinomenine over sinomenine in clinical practice, especially for inflammatory pain conditions.

In summary, this study found that *N-*demethylsinomenine, an active metabolite of sinomenine, attenuates the mechanical allodynia in mouse models of chronic neuropathic and inflammatory pain and these effects are primarily mediated by GABA_A_ receptors. Also, repeated *N-*demethylsinomenine treatment maintains its anti-allodynia throughout the experimental period, showing no trend of developing antinociception tolerance. Taken together, our findings suggest that *N-*demethylsinomenine may have potential to be developed as a valuable agent for the management of chronic pain.

## Materials and methods

### Animals

Adult male ICR mice weighing 18–22 g (Laboratory Animal Center, Nantong University, Nantong, China) were housed in groups (4–5 per cage) for at least 3 days prior to starting the experiments and acclimatized to a 12-h light/dark cycle environment with a controlled temperature of 22 ± 1 °C, and relative humidity of 50–70%. Food and water were available ad libitum except during experimental sessions. All experimental protocols were approved by the Institutional Animal Care and Use Committee, Nantong University. All procedures and the handling of animals were performed in accordance with guidelines of the International Association for the Study of Pain^39^ and the Guide for the Care and Use of Laboratory Animals (8th edition, Institute of Laboratory Animal Resources on Life Sciences, National Research Council, National Academy of Sciences, Washington, DC). Animal studies are reported in compliance with the ARRIVE guidelines^40^.

### Drugs

Sinomenine was purchased from Aladdin Reagents (Shanghai, China). *N*-demethylsinomenine was chemically synthesized in our laboratory (purity > 98% as determined by HPLC) and its chemical structure was identified to be consistent with previous reports by NMR spectroscopy^11,14^. Naltrexone hydrochloride, WAY-100635 maleate, bicuculline were obtained from Selleck Chemicals (Houston, TX, USA). WAY-100635 maleate and bicuculline were dissolved in 10% DMSO. The other drugs were dissolved in 0.9% saline. All drugs were administered intraperitoneally (i.p.) in a volume of 10 mL/kg of body weight. Complete Freund’s adjuvant (CFA) was purchased from Sigma (St. Louis, MO, USA).

### Chronic neuropathic pain model

A model of chronic neuropathic pain was induced by a chronic constriction injury (CCI) procedure, which was performed as described previously^19^. Briefly, mice were anesthetized with 2% isoflurane in oxygen at a flow rate of 3 L/min delivered via a nose cone throughout the period of surgery. After sterile preparation, an incision was made below the right hipbone, parallel to the sciatic nerve. The right common sciatic nerve was exposed at the mid-thigh level and three ligations (4.0 chromic gut, with 1 mm spacing) were loosely tied around the nerve proximal to the sciatic trifurcation until a brief twitch in the respective hind limb was observed. After nerve ligation, the incision was sutured with silk suture and a topical antibiotic was applied. Then the mice were allowed to recover in their home cages. Mice received sham procedures that the sciatic nerve was exposed without ligation served as controls.

### Chronic inflammatory pain model

Mice were temporarily anesthetized by inhalation of 3% isoflurane and then received an intraplantar injection of freshly prepared CFA (20 μL, 50% in saline) in the plantar surface of the right hind paw to induce a model of persistent inflammatory pain^41^. Mice that received an injection of the same volume of saline served as controls.

### von Frey filament test

As described in our previous study^14^, mechanical allodynia in the mice was evaluated using the von Frey filament test as previously described^42,43^. In brief, mice were placed in a clear Plexiglas chamber with an elevated wire mesh floor and allowed to acclimatize for 20 min before testing. The mechanical paw withdrawal threshold (PWT) was measured by a series of calibrated von Frey filaments (Stoelting, Kiel, WI, USA) with bending forces ranging from 0.07 to 2 g. The filaments were applied in ascending order to the mid plantar surface of each hind paw through the mesh floor. Each filament was presented vertically against the paw until it bowed slightly, and tested three times per paw with an interval of 5 s. The PWT value was defined as the minimal force to elicit paw withdrawal responses appearing at least twice in three consecutive trials. The cut-off value for this test was 2.0 g. Experimenters were well-trained to execute consistent behavioral tests and were blinded to the vehicle and treatment groups.

### Locomotor activity

The locomotor activity of mice was measured by a commercially available apparatus (YLS-1A, Shandong Academy of Medical Sciences, China), which consists of a controller unit and five separate black acrylic locomotion chambers^14^. Each chamber (12 × 15 × 15 cm) was surrounded with an array of photocell beams which link to the controller unit. Mice were individually put into these chambers in a dark environment and the spontaneous locomotor activity was measured during a 60-min test period with each count indicating one beam break by the animal.

### Experimental design

For the measurement of mechanical allodynia, the PWT was measured 24 h after CCI surgery or CFA injection and daily thereafter for 7 days. Prior to CCI surgery or CFA injection, all mice also received daily measures for 3 days to obtain the baseline value (BL) and to allow mice to habituate to the operators and procedures. For acute treatment studies, the effects of *N*-demethylsinomenine and sinomenine at three doses (10, 20, 40 mg/kg) were studied 1 day after CCI surgery or CFA injection. The PWT in all mice was measured before (0 h) and every 30 min thereafter for 4 h after the administration (i.p.) of drugs or saline (vehicle group). For repeated dosing studies, 1 day after CCI surgery or CFA injection, mice were treated with *N*-demethylsinomenine (20 and 40 mg/kg) once daily for 2 weeks. The PWT was measured daily before (0 h) and 1.5 h after *N*-demethylsinomenine administration. In an effort to examine the pharmacological mechanisms mediating *N*-demethylsinomenine induced anti-allodynia against CCI-induced neuropathic pain and CFA-induced inflammatory pain, we investigated whether pretreatment with different antagonists including bicuculline (a selective GABA_A_ receptor antagonist), WAY100635 (serotonin 5-HT_1A_ receptor antagonist) or naltrexone (opioid receptor antagonist) affected the antinociception of 40 mg/kg *N*-demethylsinomenine. For antagonist studies, 1 day after CCI surgery or CFA injection, different antagonists were administered 10 min prior to 40 mg/kg *N*-demethylsinomenine administration respectively and the PWT was measured thereafter every 30 min for 4 h. For locomotor activity test, the doses of 10–40 mg/kg sinomenine and *N*-demethylsinomenine were studied to observe the potential sedative effect in healthy mice. The drug was administered 60 min before the test sessions because this pretreatment time was adequate for the drug to produce significant anti-allodynic effects. For all the studies, a blind design was strictly followed so that different experimenters performed drug treatments and behavioral tests.

### Data analyses

All data were expressed as mean ± standard error of the mean (S.E.M.) and were analyzed by the GraphPad Prism 5.01 software (San Diego, CA, USA). For mechanical allodynia test, the PWT (g) was plotted as a function of time (h or days). The statistical differences between groups were analyzed by two-way analysis of variance (ANOVA) with repeated measures (Time × Treatment) followed by Bonferroni post hoc analysis. The antinociceptive effect of each drug dose was quantified for each animal as the percentage of the maximum possible effect (% MPE) according to the following formula: % MPE = [(post-drug PWT − pre-drug PWT)/(normal baseline PWT − pre-drug PWT)] × 100. The dose–effect curves for *N*-demethylsinomenine and sinomenine were constructed by plotting the effect (% MPE) of each dose and their individual ED_50_ [95% confidence limits (CL)] values were calculated respectively using nonlinear regression method by the GraphPad Prism 5.01 software (San Diego, CA, USA). A value of *P* < 0.05 was considered statistically significant for all analyses.

## Supplementary Information

Supplementary Information.

## Abbreviations

CCI: Chronic constriction injury
CFA: Complete Freund’s adjuvant
GABA: γ-Aminobutyric acid
PWT: Paw withdrawal threshold
MPE: Maximum possible effect
CL: Confidence limits
NSAID: Nonsteroidal anti-inflammatory drug
NMDA: *N*-Methyl-d-aspartic acid
mTOR: Mammalian target of rapamycin
5-HT: 5-Hydroxytryptamine
ANOVA: Analysis of variance
HPLC: High performance liquid chromatography
NMR: Nuclear magnetic resonance
SEM: Standard error of the mean
