# Supplementary material for: N-Demethylsinomenine, an active metabolite of sinomenine, attenuates chronic neuropathic and inflammatory pain in mice
Source: Sci Rep. 2021 Apr 29;11:9300. doi: 10.1038/s41598-021-88521-z (PMC8085237; doi:10.1038/s41598-021-88521-z)
Supplement: Supplementary file 1 — Supplementary Information. [file 41598_2021_88521_MOESM1_ESM.ppt]

## Slide 1
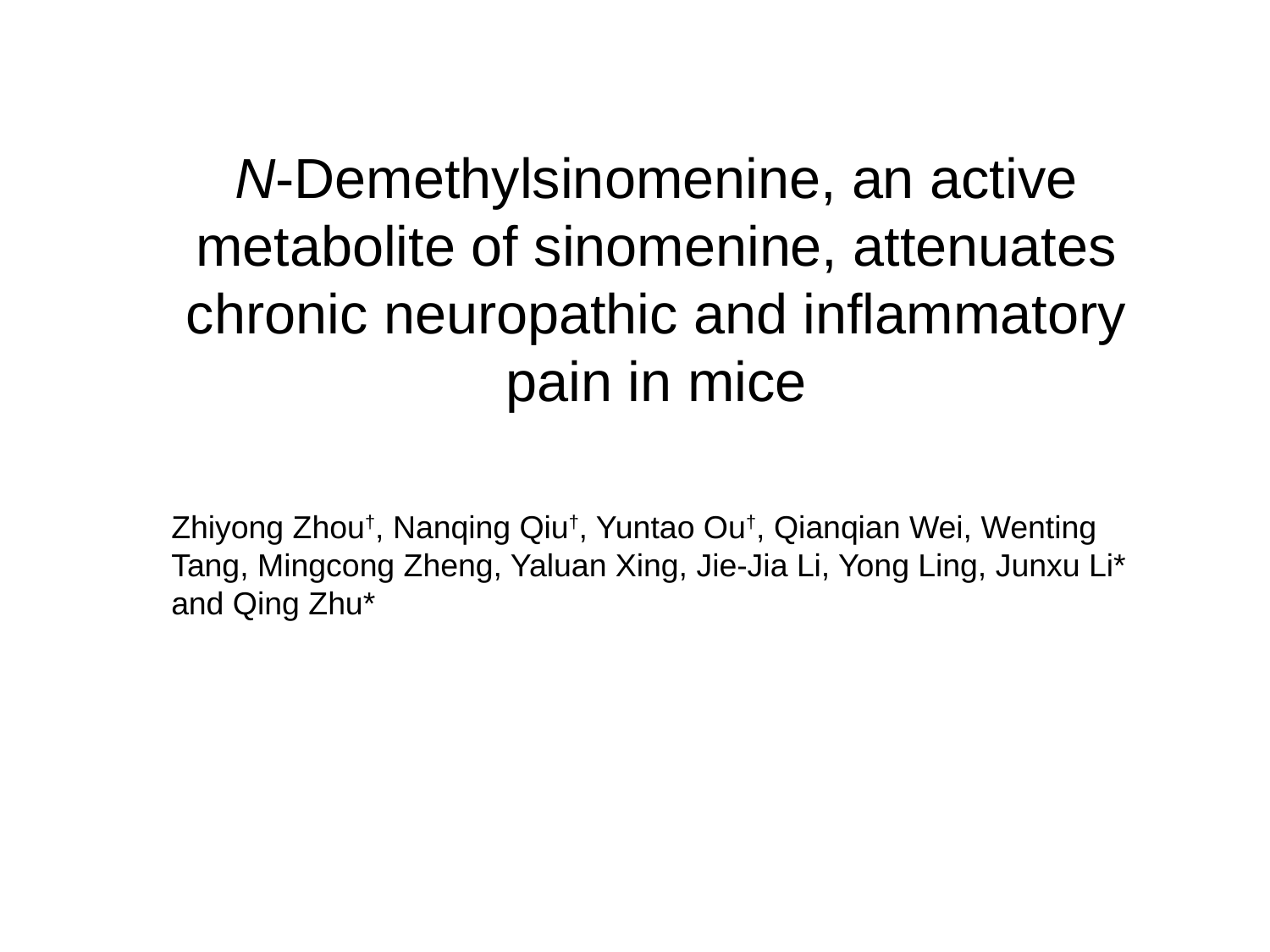

# N-Demethylsinomenine, an active metabolite of sinomenine, attenuates chronic neuropathic and inflammatory pain in mice
Zhiyong Zhou†, Nanqing Qiu†, Yuntao Ou†, Qianqian Wei, Wenting Tang, Mingcong Zheng, Yaluan Xing, Jie-Jia Li, Yong Ling, Junxu Li* and Qing Zhu*

## Slide 2
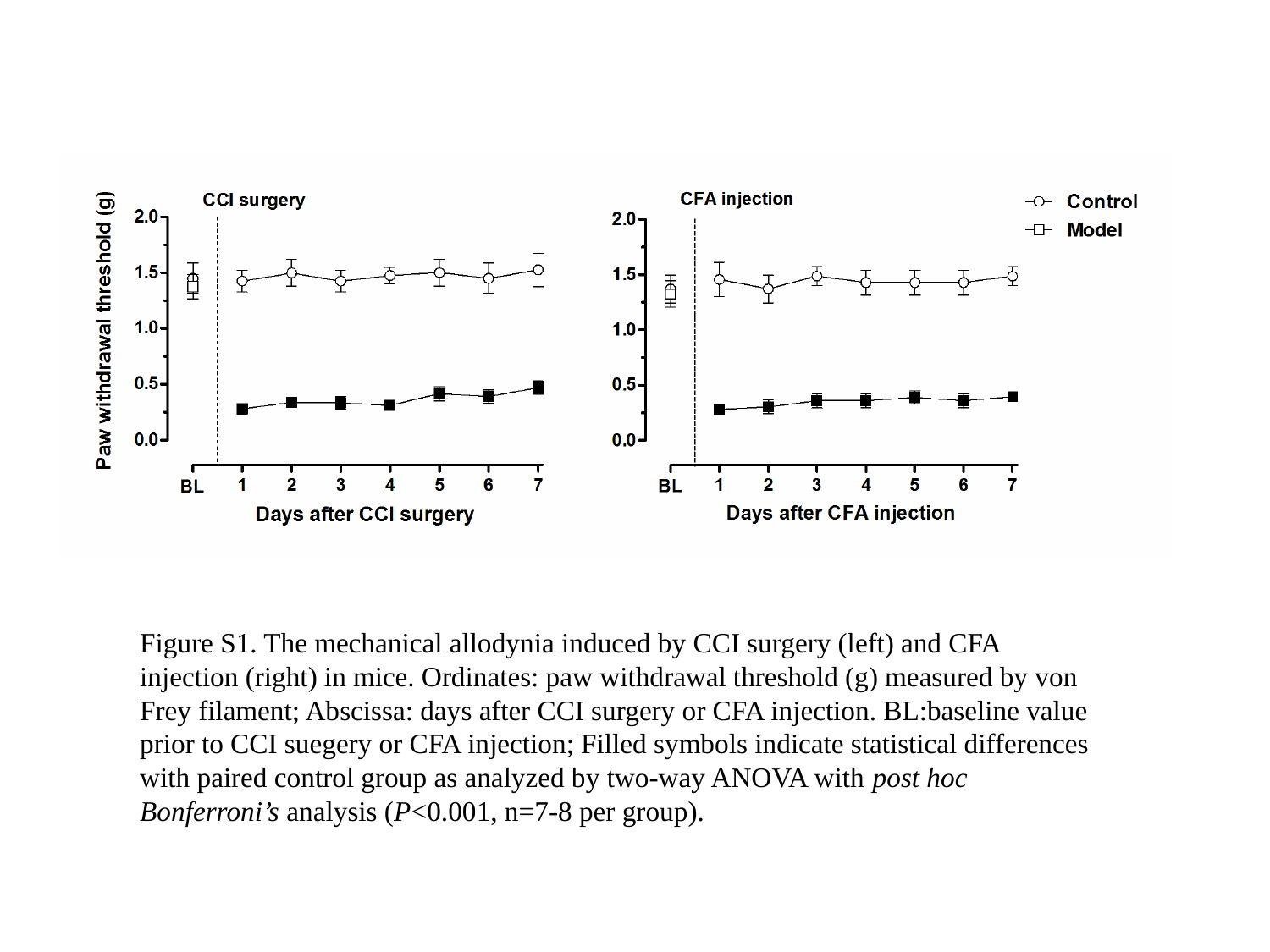

Figure S1. The mechanical allodynia induced by CCI surgery (left) and CFA injection (right) in mice. Ordinates: paw withdrawal threshold (g) measured by von Frey filament; Abscissa: days after CCI surgery or CFA injection. BL:baseline value prior to CCI suegery or CFA injection; Filled symbols indicate statistical differences with paired control group as analyzed by two-way ANOVA with post hoc Bonferroni’s analysis (P<0.001, n=7-8 per group).

## Slide 3
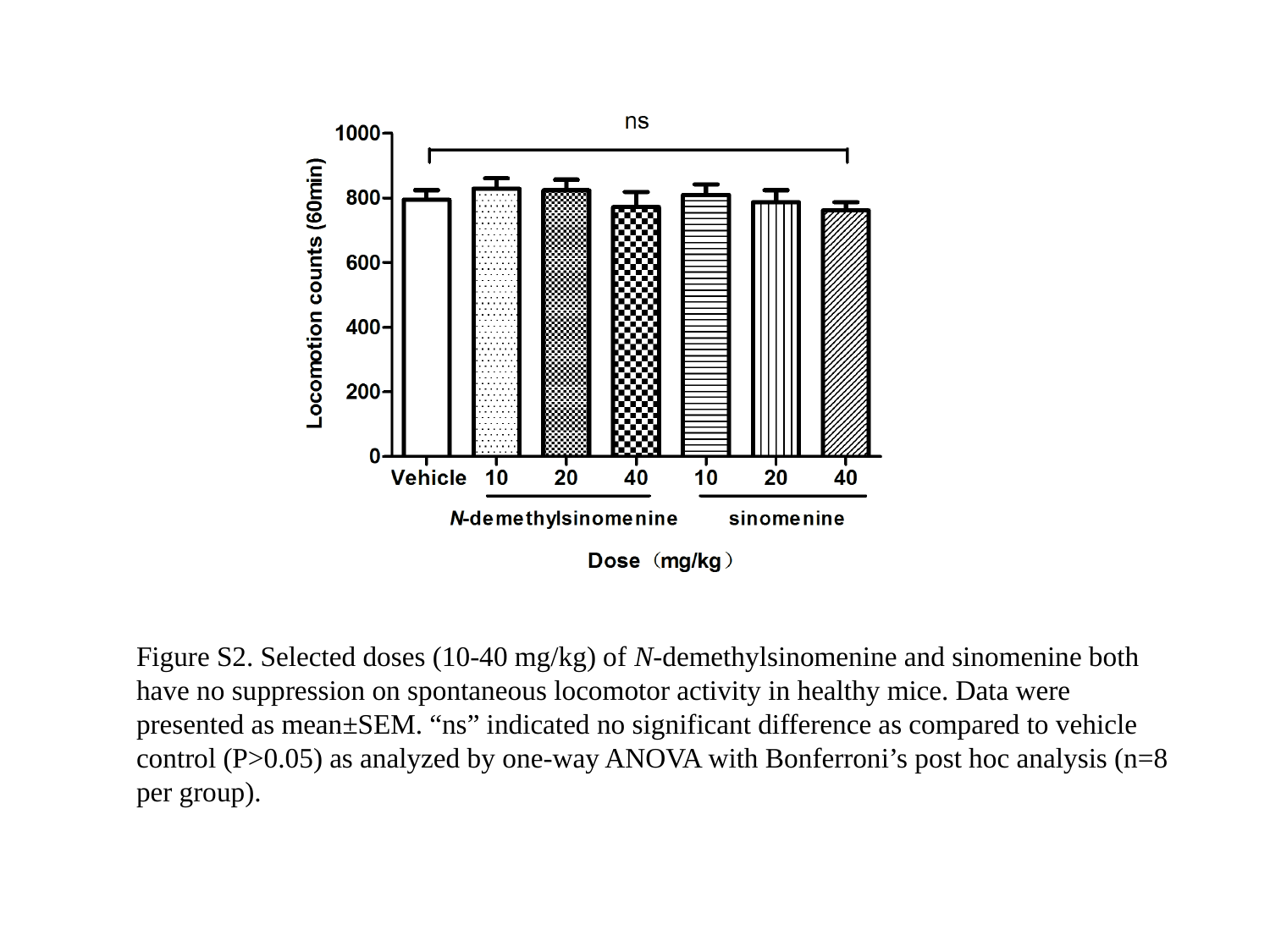

Figure S2. Selected doses (10-40 mg/kg) of N-demethylsinomenine and sinomenine both have no suppression on spontaneous locomotor activity in healthy mice. Data were presented as mean±SEM. “ns” indicated no significant difference as compared to vehicle control (P>0.05) as analyzed by one-way ANOVA with Bonferroni’s post hoc analysis (n=8 per group).

## Slide 4
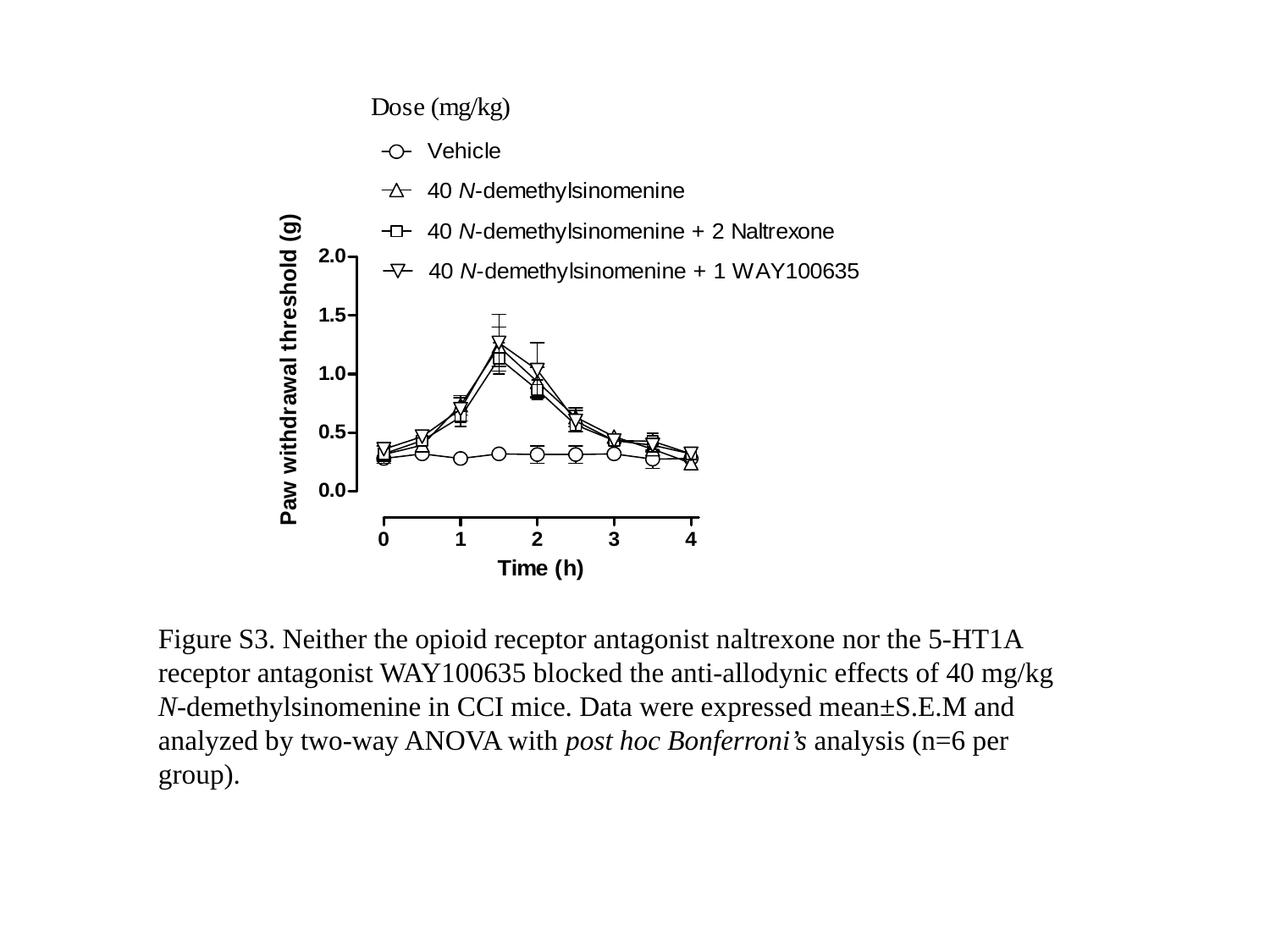

Figure S3. Neither the opioid receptor antagonist naltrexone nor the 5-HT1A receptor antagonist WAY100635 blocked the anti-allodynic effects of 40 mg/kg N-demethylsinomenine in CCI mice. Data were expressed mean±S.E.M and analyzed by two-way ANOVA with post hoc Bonferroni’s analysis (n=6 per group).
